# Multiple reports on the causal relationship between various chronic pain and gut microbiota: a two-sample Mendelian randomization study

**DOI:** 10.3389/fnins.2024.1369996

**Published:** 2024-04-17

**Authors:** Yuxin Cai, Shuyang Wen, Jinjing Hu, Ziyi Wang, Guozhi Huang, Qing Zeng, Jihua Zou

**Affiliations:** ^1^Department of Rehabilitation Medicine, Zhujiang Hospital, Southern Medical University, Guangzhou, China; ^2^School of Rehabilitation Medicine, Southern Medical University, Guangzhou, China; ^3^School of Nursing, Southern Medical University, Guangzhou, China; ^4^The First School of Clinical Medicine, Southern Medical University, Guangzhou, China; ^5^Faculty of Health and Social Sciences, Hong Kong Polytechnic University, Kowloon, Hong Kong SAR, China

**Keywords:** gut microbiota, chronic pain, Mendelian randomization, genetics, single-nucleotide polymorphisms (SNPs)

## Abstract

**Background:**

Previous evidence suggests a link between gut microbiota and chronic pain, but the causal relationship is not yet fully understood.

**Methods:**

We categorized gut microbiota based on phylum, class, order, family, and genus levels and gathered pain-related information from the UKB and FinnGen GWAS project. Then, we conducted MR analysis to explore the potential causal relationship between gut microbiota and chronic pain at 12 specific locations.

**Results:**

We have discovered a direct connection between genetic susceptibility in the gut microbiota (gut metabolites) and pain experienced at 12 specific locations. Notably, Serotonin (5-HT) and Glycine were found to be associated with a higher risk of pain in the extremities. On the other hand, certain microbial families and orders were found to have a protective effect against migraines. Specifically, the family Bifidobacteriaceae (IVW, FDR *p* = 0.013) was associated with a lower risk of migraines. Furthermore, the genus Oxalobacter (IVW, FDR *p* = 0.044) was found to be linked to an increased risk of low back pain. Importantly, these associations remained significant even after applying the Benjamini-Hochberg correction test. Our analysis did not find any heterogeneity in the data (*p* > 0.05), as confirmed by the Cochrane’s *Q*-test. Additionally, both the MR-Egger and MR-PRESSO tests indicated no significant evidence of horizontal pleiotropy (*p* > 0.05).

**Conclusion:**

Our MR analysis demonstrated a causal relationship between the gut microbiota and pain, highlighting its potential significance in advancing our understanding of the underlying mechanisms and clinical implications of microbiota-mediated pain.

## 1 Introduction

Pain is defined as unpleasant sensory and emotional experience associated with, or resembling that associated with, actual or potential tissue damage (Raja et al., 2020).

It is the primary reason why individuals seek medical care and is a major contributor to disability worldwide. Chronic pain, which refers to persistent or recurring pain lasting over 3 months, is particularly burdensome, affecting more than 30% of the global population and causing significant personal and financial challenges. Chronic pain has also been associated with reduced life expectancy, even after accounting for factors such as higher rates of depression, suicide, and opioid use (Cohen et al., 2021). It is important to note that the molecular and cellular mechanisms underlying chronic pain are still not fully understood. Additionally, there remains a lack of safe, well-tolerated, and effective treatments for this condition (Eccleston et al., 2021).

As the most intricate and populous microecosystem in our body, the intestinal microbiota consists of bacteria, archaea, viruses, and fungi. Trillions of diverse bacterial species colonize the gastrointestinal tract in a spatially organized manner, containing over 200 times the number of genes found in the human genome (Backhed et al., 2005; Sender et al., 2016). Maintaining a good gut microbiota diversity is critical for normal life, and its changes (dysbiosis) affect the gut-brain axis, leading to a variety of neurological diseases, including Alzheimer’s disease (AD), Parkinson’s disease (PD), traumatic brain injury, depression, and chronic pain (Sharon et al., 2016; Cryan et al., 2020; Sorboni et al., 2022). Changes in the gut microbiota in patients suffer from different types and regions of chronic pain, including visceral pain, inflammatory pain, headache, neuropathic pain, and chronic generalized pain (Newlove-Delgado et al., 2019; Guida et al., 2020). Meanwhile, some studies also suggest that the gut microbiota may play a causal role in pain. Targeting the gut microbiota by dietary and pharmacologic abiotic intervention may represent a novel therapeutic strategy for treating chronic pain (Guo et al., 2019). In the correlation study showed that the change of intestinal microbiota is associated with human chronic pain and some postoperative results, may mediate the pathogenesis of chronic pain, the future is the urgent need to a more comprehensive understanding of the pathogenesis of individual bacterial taxa in pain, identification and isolation of chronic pain common bacterial taxa and specific diagnosis of cell taxa (Minerbi and Shen, 2022).

In conclusion, we believe that two possibilities should not be overlooked: chronic pain may lead to intestinal dysregulation, and the intestinal microbiota may serve as a potential modulator of chronic pain. However, the intestinal microbiota is a functionally complex entity within an ecosystem, and the causal relationship between the intestinal microbiota, microbial metabolites, and chronic pain remains to be established.

Current clinical studies are mostly observational, and their results are susceptible to confounding factors, eliminating some disadvantages such as limited sample size and prospective design. Hindered our study of the prevention and treatment of chronic pain attacks. Traditionally, well-designed randomized controlled trials have been the gold standard to infer causal relationships between the gut microbiota and chronic pain; however, they are difficult to implement due to ethical and legal limitations. Mendelian randomization as an emerging method is used to determine potential causal relationships between exposure factors and outcomes. Using genetic variants as an unconfounding proxy for exposure, specific single-nucleotide polymorphisms were used as instrumental variables (IVs) (Hemani et al., 2018). Lack of alleles, this design is unlikely to be confused or influenced by reverse causality due to the random distribution of alleles during gamete formation. Based on the strengths of the study design, MR can well reveal the causal effects of exposure and outcome. With the flourishing of publicly available large sample size GWAS data, obtaining higher statistical power is more effective.

Previously, a MR design has been used to explore the causal relationship of gut microbiota with many diseases, such as major depressive disorder (MDD) (Amin et al., 2023), AD (Zhuang et al., 2020), diabetes (Yuan et al., 2023), ischemic stroke (Meng et al., 2023), etc. However, there has not been a specific study investigating the impact of intestinal microbiota on chronic pain in different parts of the body. Therefore, this Mendelian analysis examines the potential causal relationship between individual bacterial taxa and chronic pain at specific sites, involving 212 bacterial taxa and 12 distinct sites of chronic pain. Metabolites play a crucial role in connecting the gut microbiota and the central nervous system. Hence, the analysis also explores potential associations between metabolites and chronic pain, aiming to provide novel insights into the gut-brain axis and interventions for chronic pain.

## 2 Materials and methods

### 2.1 Study design

**Figure 1:**
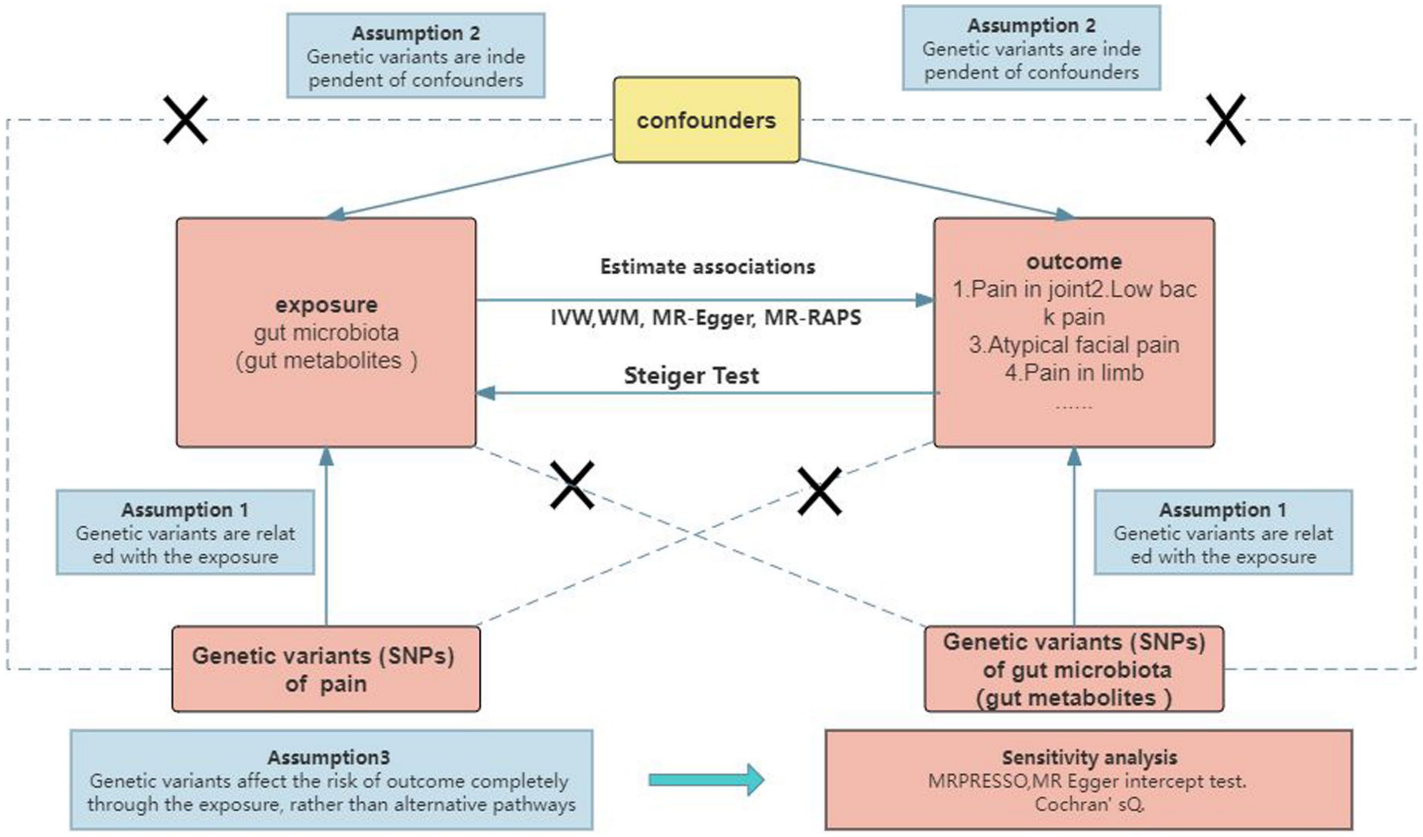
Study design.

To investigate the causal relationship between gut microbiota (specifically gut microbial metabolites) and chronic pain at 12 specific sites, a bidirectional two-sample MR approach was employed. Summary statistics from large GWAS were utilized, as depicted in Figure 1 and Supplementary Table S1. The ethical approval for each GWAS included in this study can be found in the original articles. The study followed a standard two-sample framework in accordance with Burgess’s guidelines and was reported following the STROBE-MR statement (Burgess et al., 2019). Ethical considerations were taken into account in this study. The analysis was conducted using summary-level data that had already been published and made publicly available. Therefore, no additional ethical approval or informed consent was required for this study. It is important to note that ethical approval had been obtained for all original studies included in the analysis.

### 2.2 Data sources and study population

#### 2.2.1 Data sources of gut microbiota and gut microbial metabolites

A total of 211 gut microbiomes, spanning from the genus to phylum level, were identified in this study. To ensure reproducibility, all bacterial traits were analyzed using three 16S rRNA regions and rarefied to 10,000 reads. This rarefaction method allowed for consistent comparison across samples. The analysis resulted in the identification of 131 genera, 16 classes, 35 families, 20 orders, and 9 phyla within the gut microbiome. To account for potential confounding factors, such as sex and age, these covariates were adjusted for in all cohorts (Kurilshikov et al., 2021). For more detailed information regarding the composition and characteristics of the gut microbiome, refer to Supplementary Table S1.

#### 2.2.2 Data sources of pain

The genetic associations for pain at various body sites were obtained from the Large-scale GWAS meta-analysis, the United Kingdom Biobank, and the FinnGen consortium R9 (Kurki et al., 2023). Specifically, we extracted GWAS summary statistics for pain at 12 specific sites, namely headache, ocular pain, facial pain, neck or shoulder pain, back pain, knee pain, hip pain, limb pain, joint pain, fibromyalgia, pain in thoracic spine, and pain involving the limbs, back, neck, and abdomen. Supplementary Table S1 provides detailed information on the specific data sources for our study, the cohorts involved, genotypes, definitions of endpoints, and association tests.

### 2.3 Selection of IVs

To ensure the reliability and accuracy of our findings, we performed quality checks on the IVsto identify suitable IVs. The selection of IVs adhered to the following principles: (1) The genetic variants should be associated with the exposure of interest, (2) The genetic variants should be independent of any confounding factors, and (3) The genetic variants should exert their effects on the outcome solely through the exposure, rather than through alternative pathways (Burgess et al., 2019). Given the limited number of IVs that met the genome-wide statistical significance threshold (*p* < 5 × 10–8), we decided to employ a locus-wide significance threshold of *p* < 1 × 10–5 to obtain a more comprehensive set of IVs (Auton et al., 2015; Jia et al., 2019). To account for any potential effects of linkage disequilibrium (LD), we applied a clumping method with an r2 threshold of 0.001 and a distance threshold of 10,000 base pairs.

### 2.4 Statistical analysis

To explore the causal relationship between microbiome signatures and chronic pain across different body sites, we performed MR analyses. We employed five commonly used MR methods, namely inverse-variance weighted (IVW) (Burgess et al., 2013),^1^ weighted mode (Hartwig et al., 2017),^2^ MR-Egger3 (Bowden et al., 2015),^3^ weighted median (WME) (Bowden et al., 2016),^4^ and simple mode.^5^ Among these methods, IVW was considered slightly more powerful than the others under specific conditions, so the primary analysis relied on IVW, while the other methods were used as supplementary analyses (Bowden et al., 2016), To assess the robustness of the significant results, sensitivity analysis methods such as MR-Egger, weighted median, weighted mode, and simple mode were employed.

To detect potential heterogeneity and evaluate the validity of instruments, we conducted a heterogeneity test using Cochran’s *Q*-test^6^ and the two-sample MR package (Greco et al., 2015). A *Q*-value greater than the number of instruments minus one indicates evidence of heterogeneity and invalid instruments. Alternatively, when the *Q*-test yield a *p*-value less than 0.05, it suggests the presence of heterogeneity. To investigate the potential causal impact of pain on the identified significant bacterial genera, we performed a reverse MR analysis. This analysis utilized IVs associated with pain as IVs, with pain as the exposure and the identified causal bacterial genus as the outcome. For this analysis, we employed the MR Steiger directionality test (Hemani et al., 2017).

To determine the statistical significance of the MR effect estimates, we used a Benjamini-Hochberg false discovery rate (FDR) threshold of less than 5% to account for multiple comparisons. All analyses were conducted using “TwoSampleMR,” “jvenn (Bardou et al., 2014),” “MRPRESSO,” “frostplot,” and “ggplot2” in the R software (R version 4.3.02023-04-21 ucrt).

## 3 Results

### 3.1 IVs selection

Initially, we identified a total of 13,749 IVs associated with gut microbiota and 66 SNPs associated with gut microbial metabolites as potential IVs from large-scale genome-wide association studies (GWAS). After excluding palindromic SNPs, we further refined this set of IVs. Among them, there were 211 bacterial traits, which were classified into five biological categories: phylum (245 SNPs), class (42 SNPs), order (523 SNPs), family (803 SNPs), and genus (2,703 SNPs). Additionally, we identified 9 gut microbial metabolites, including 3-hydroxybutyrate (BHBA) (10 SNPs), Tryptophan (159 SNPs), Tyrosine (42 SNPs), Phenylalanine (40 SNPs), Glycine (100 SNPs), propionic acid (22 SNPs), 5-HT (15 SNPs), and Trimethylamino oxide (21 SNPs).

After performing clumping and harmonization procedures, a total of 5,078 SNPs with a significance level of *p* < 1 × 10–5 were selected as IVs. We systematically collected detailed information about the key features of these SNPs, including the effect allele, other allele, beta coefficient, standard error, and *p*-value, for further analysis. Detailed information on the instruments for gut metabolites can be found in Supplementary Table S7.

### 3.2 The causal associations between gut microbiota and pain

#### 3.2.1 Headache

The results obtained from the IVW test revealed significant associations between genetically predicted relative abundance of certain bacterial taxa and the risk of headache. The estimated OR and 95% confidence intervals (CI) for these associations were as follows:

- Family.ClostridialesvadinBB60gr: OR = 0.718, 95% CI: 0.972 0.53, *p* = 0.032;
- Family.Alcaligenaceae: OR = 0.597, 95% CI: 0.903 0.394, *p* = 0.015;
- Family.Bifidobacteriaceae: OR = 0.823, 95% CI: 0.901 0.752, *p* = 0.000025;
- Order.Burkholderiales: OR = 0.56, 95% CI: 0.35 0.88, *p* = 0.012;
- Class.Betaproteobacteria: OR = 0.59, 95% CI: 0.38 0.91, *p* = 0.018;
- Genus.Coprococcus1: OR = 0.59, 95% CI: 0.39 0.89, *p* = 0.011;
- Order.Bifidobacteriales: OR = 0.823, 95% CI: 0.901 0.752, *p* = 0.000025;
- Genus.Eubacteriumcoprostanolig: OR = 0.852, 95% CI: 0.96 0.756, *p* = 0.009;
- Genus.Eubacteriumrectalegroup: OR = 0.877, 95% CI: 0.985 0.782, *p* = 0.026;
- Genus.Bifidobacterium: OR = 0.864, 95% CI: 0.97 0.769, *p* = 0.013.

These findings suggest a negative association between the genetically influenced abundance of these bacterial taxa and the risk of headache. Conversely, the genetic predisposition to headache was positively associated with the relative abundance of the following taxa:

- Phylum.Actinobacteria: OR = 1.643, 95% CI: 2.472 1.092, *p* = 0.017;
- Genus.Oxalobacter: OR = 1.069, 95% CI: 1.137 1.006, *p* = 0.032;
- Genus.Victivallis: OR = 1.06, 95% CI: 1.121 1.002, *p* = 0.041.

**Figure 2:**
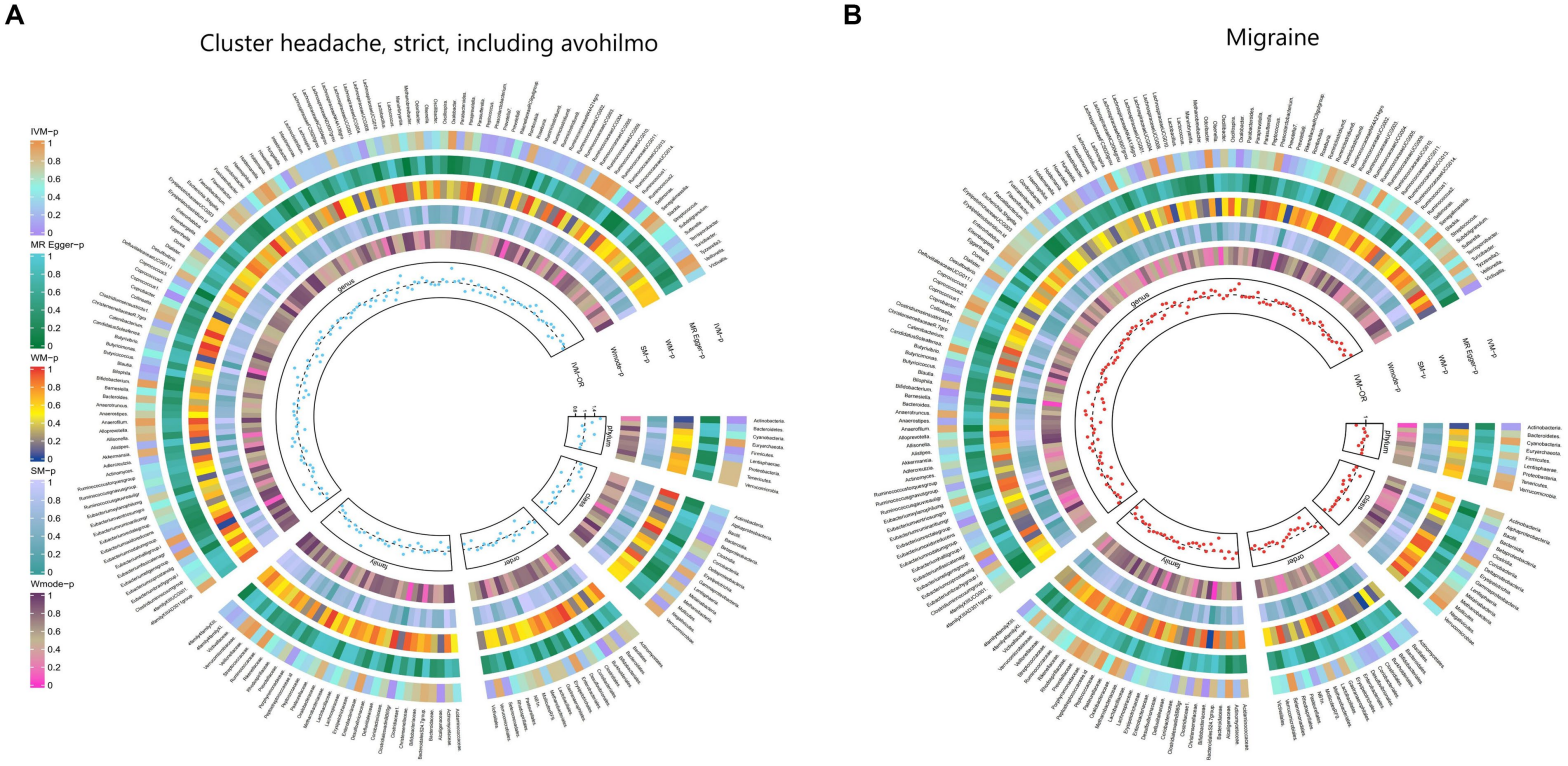
From outside to inside, the *p-*values of IVW, MR Egger, WM, SM, and Wmode represented, respectively. IVW, inverse variance weighted; WM, weighted median; SM, simple mode; Wmode weighted mode. **(A)** Causal effect of the gut microbiome on headache. **(B)** Causal effect of the gut microbiome on migraine in FinnGen Biobank based on MR analyses.

Furthermore, Figure 2 and Supplementary Table S8 provided evidence of the causal effects of 196 gut microbiomes on the occurrence of headache.

#### 3.2.2 Ocular pain

The IVW test results indicated significant associations between genetically predicted relative abundance of certain bacterial taxa and the risk of ocular pain. The estimated OR and 95%CI for these associations were as follows:

- Genus.Oxalobacter: OR = 0.815, 95% CI: 0.967 0.687, *p* = 0.019;
- Order.Clostridiales: OR = 0.701, 95% CI: 0.997 0.493, *p* = 0.048;
- Class.Clostridia: OR = 0.702, 95% CI: 0.998 0.494, *p* = 0.049.

These findings suggest a negative association between the genetically influenced abundance of these bacterial taxa and the risk of ocular pain. On the other hand, the genetic predisposition to ocular pain was positively associated with the relative abundance of the following taxa:

- Genus.Intestinimonas: OR = 1.333, 95% CI: 1.675 1.06, *p* = 0.014;
- Genus.RuminococcaceaeUCG014: OR = 1.355, 95% CI: 1.733 1.059, *p* = 0.016;
- Order.Bacillales: OR = 1.218, 95% CI: 1.446 1.026, *p* = 0.024;
- Genus.Catenibacterium: OR = 1.358, 95% CI: 1.776 1.038, *p* = 0.025.

**Figure 3:**
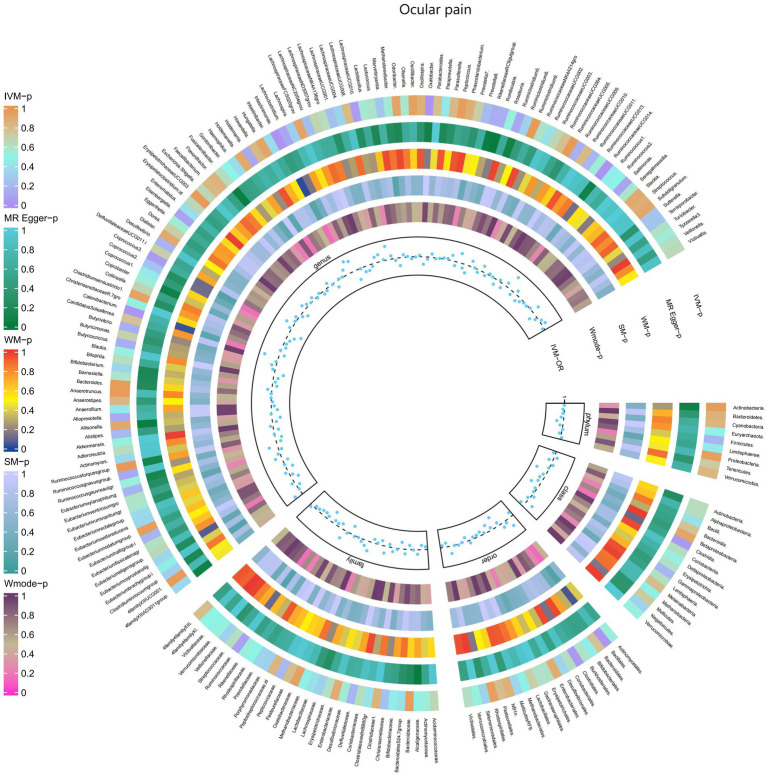
Causal effect of the gut microbiome on Ocular pain in FinnGen Biobank based on MR analyses. From outside to inside, the *p*-values of IVW, MR Egger, WM, SM, and Wmode represented, respectively. IVW, inverse variance weighted; WM, weighted median; SM, simple mode; Wmode weighted mode.

Furthermore, Figure 3 and Supplementary Table S9 provided evidence of the causal effects of 196 gut microbiomes on the occurrence of ocular pain.

#### 3.2.3 Facial pain

The IVW test results demonstrated significant associations between genetically predicted relative abundance of certain bacterial taxa and the risk of facial pain. The estimates, expressed as OR with corresponding 95%CI, were as follows:

- Genus.Phascolarctobacterium: OR = 0.59, 95% CI: 0.861 0.405, *p* = 0.006;
- Order.NB1n: OR = 0.779, 95% CI: 0.957 0.634, *p* = 0.018;
- Genus.Alloprevotella: OR = 0.711, 95% CI: 0.923 0.547, *p* = 0.011.

These findings indicate a negative association between genetically influenced abundance of these bacterial taxa and the risk of facial pain. Conversely, the genetic predisposition to facial pain was positively associated with the relative abundance of the following taxa:

- Genus.RuminococcaceaeUCG005: OR = 1.435, 95% CI: 1.966 1.047, *p* = 0.025;
- Phylum.Bacteroidetes: OR = 1.606, 95% CI: 2.457 1.049, *p* = 0.029;
- Genus.Anaerofilum: OR = 1.29, 95% CI: 1.646 1.011, *p* = 0.041.

**Figure 4:**
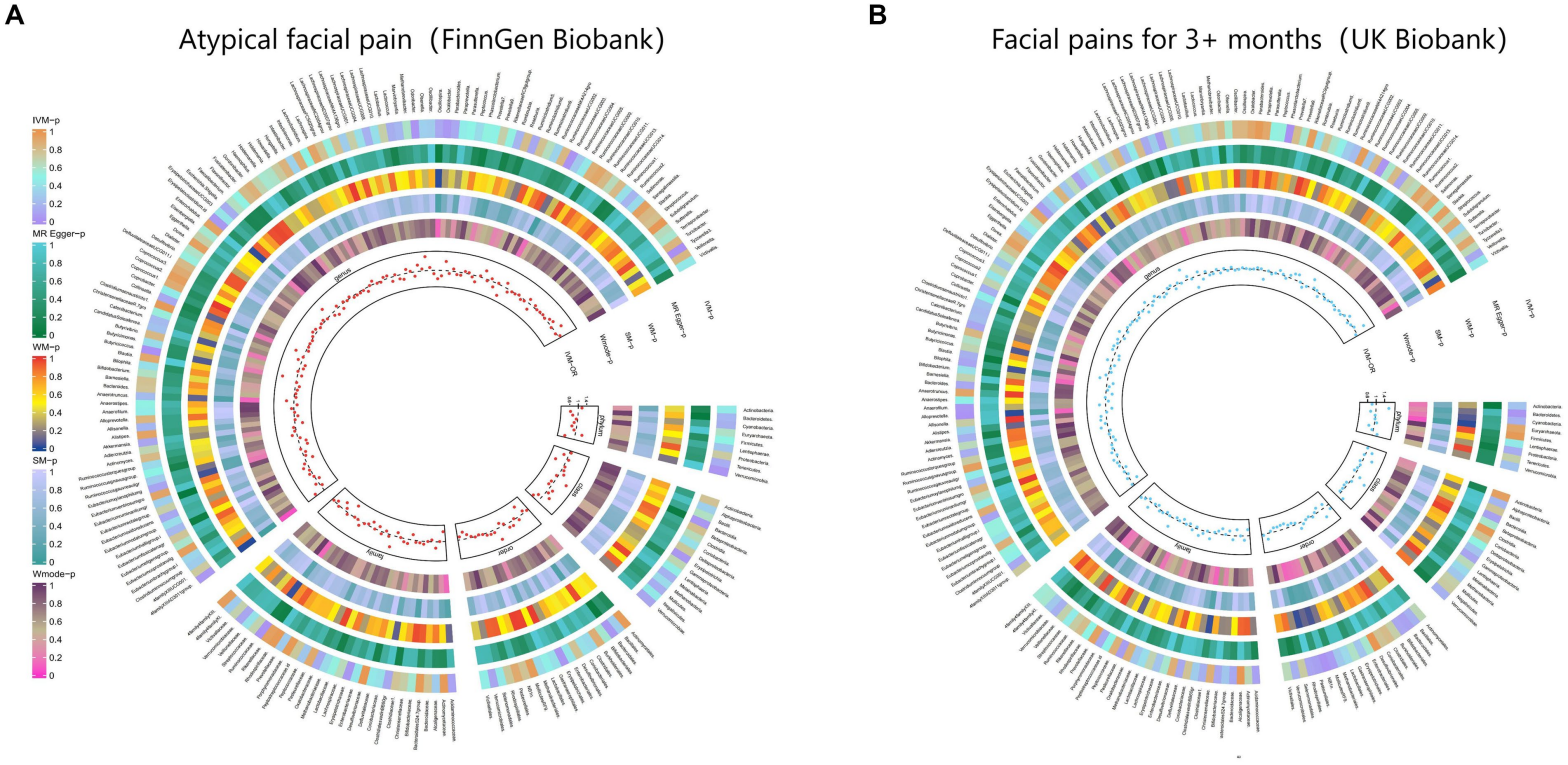
From outside to inside, the *p*-values of IVW, MR Egger, WM, SM, and Wmode represented, respectively. IVW, inverse variance weighted; WM, weighted median; SM, simple mode; Wmode weighted mode. **(A)** Causal effect of the gut microbiome on Atypical facial pain (FinnGen Biobank). **(B)** Causal effect of the gut microbiome on Facial pains (UK Biobank) based on MR analyses.

Additionally, Figure 4 and Supplementary Table S10 provided evidence of the causal effects of 196 gut microbiomes on the occurrence of facial pain.

#### 3.2.4 Neck or shoulder pain

The IVW test results revealed significant associations between genetically predicted relative abundance of certain bacterial taxa and the risk of neck or shoulder pain. The estimates, expressed as OR with corresponding 95%CI, were as follows:

- Family.Actinomycetaceae: OR = 0.976, 95% CI: 0.997 0.955, *p* = 0.027;
- Order.Actinomycetales: OR = 0.976, 95% CI: 0.997 0.955, *p* = 0.027;
- Genus.LachnospiraceaeUCG010: OR = 0.976, 95% CI: 0.995 0.959, *p* = 0.012;
- Genus.Escherichia.Shigella: OR = 0.977, 95% CI: 0.995 0.96, *p* = 0.012;
- Genus.Faecalibacterium: OR = 0.98, 95% CI: 0.998 0.962, *p* = 0.031.

These findings indicate a negative association between genetically influenced abundance of these bacterial taxa and the risk of neck or shoulder pain. Conversely, the genetic predisposition to neck or shoulder pain was positively associated with the relative abundance of the following taxa:

- Family.Rhodospirillaceae: OR = 1.017, 95% CI: 1.033 1.001, *p* = 0.041;
- Order.Gastranaerophilales: OR = 1.016, 95% CI: 1.029 1.003, *p* = 0.015;
- Genus.Methanobrevibacter: OR = 1.016, 95% CI: 1.029 1.002, *p* = 0.023;
- Genus.Eubacteriumnodatumgroup: OR = 1.012, 95% CI: 1.022 1.001, *p* = 0.026.

**Figure 5:**
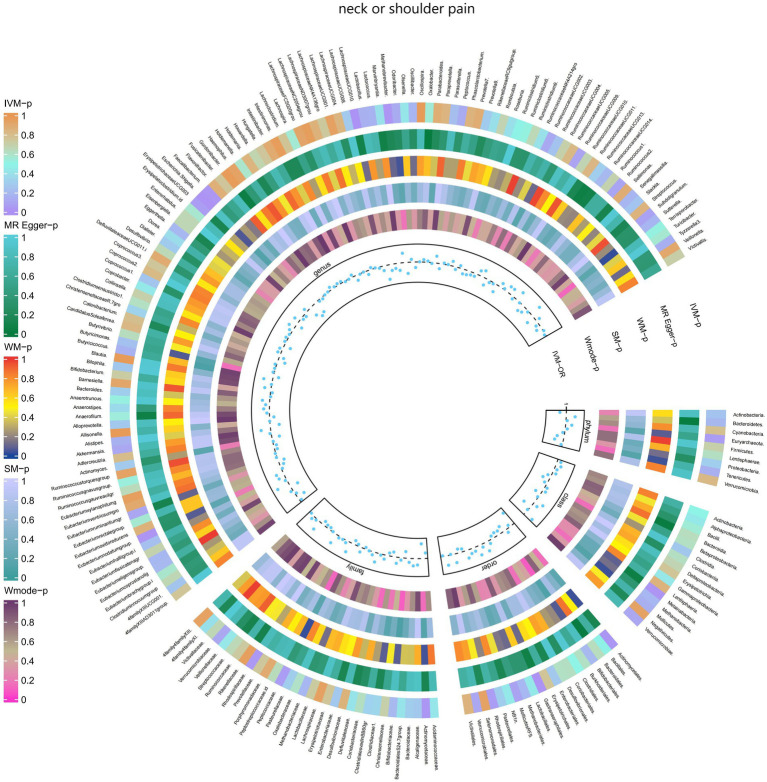
Causal effect of the gut microbiome on neck or shoulder pain (UK Biobank) on MR analyses. From outside to inside, the *p-*values of IVW, MR Egger, WM, SM, and Wmode represented, respectively. IVW, inverse variance weighted; WM, weighted median; SM, simple mode; Wmode weighted mode.

Additionally, Figure 5 and Supplementary Table S11 provided evidence of the causal effects of 196 gut microbiomes on the occurrence of neck or shoulder pain.

#### 3.2.5 Back pain

The IVW test results revealed significant associations between genetically predicted relative abundance of certain bacterial taxa and the risk of back pain. The estimates, expressed as OR with corresponding 95%CI, were as follows:

- Phylum.Firmicutes: OR = 0.925, 95% CI: 0.987 0.866, *p* = 0.018;
- FamilyXIII: OR = 0.893, 95% CI: 0.995 0.802, *p* = 0.04;
- Family.Streptococcaceae: OR = 0.906, 95% CI: 0.99 0.83, *p* = 0.029;
- Genus.Lactobacillus: OR = 0.941, 95% CI: 0.999 0.885, *p* = 0.047;
- Genus.Roseburia: OR = 0.9, 95% CI: 0.977 0.829, *p* = 0.012;
- Genus.Olsenella: OR = 0.932, 95% CI: 0.978 0.889, *p* = 0.004;
- Genus.RuminococcaceaeUCG011: OR = 0.949, 95% CI: 0.992 0.907, *p* = 0.021;
- Genus.Eubacteriumrectalegroup: OR = 0.897, 95% CI: 0.97 0.83, *p* = 0.006;
- Genus.Collinsella: OR = 0.889, 95% CI: 0.965 0.819, *p* = 0.005;
- Genus.Eisenbergiella: OR = 0.937, 95% CI: 0.997 0.881, *p* = 0.038;
- Genus.Streptococcus: OR = 0.915, 95% CI: 0.996 0.84, *p* = 0.04.

These findings suggest a negative association between genetically influenced abundance of these bacterial taxa and the risk of back pain. On the other hand, the genetic predisposition to back pain was positively associated with the relative abundance of the following taxa:

- Family.Prevotellaceae: OR = 1.085, 95% CI: 1.174 1.003, *p* = 0.042;
- Genus.Oxalobacter: OR = 1.066, 95% CI: 1.11 1.023, *p* = 0.002;
- Genus.Ruminiclostridium6: OR = 1.073, 95% CI: 1.144 1.007, *p* = 0.029;
- Genus.RuminococcaceaeUCG005: OR = 1.063, 95% CI: 1.129 1, *p* = 0.049;
- Genus.Tyzzerella3: OR = 1.047, 95% CI: 1.095 1.002, *p* = 0.04;
- Genus.Eubacteriumfissicatenagr: OR = 1.066, 95% CI: 1.126 1.01, *p* = 0.022;
- Genus.Allisonella: OR = 1.06, 95% CI: 1.103 1.018, *p* = 0.005.

**Figure 6:**
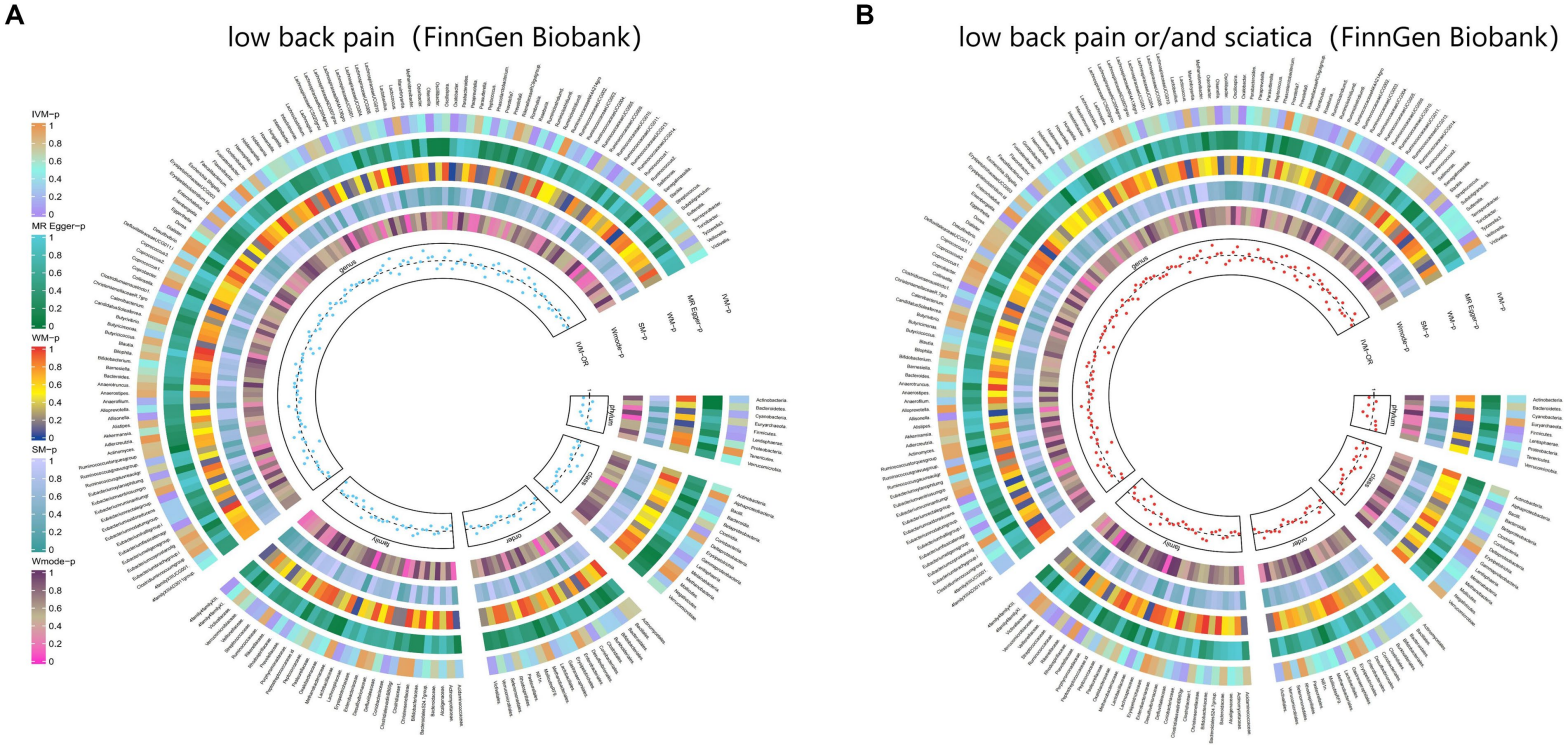
From outside to inside, the *p*-values of IVW, MR Egger, WM, SM, and Wmode represented, respectively. IVW, inverse variance weighted; WM, weighted median; SM, simple mode; Wmode weighted mode. **(A)** Causal effect of the gut microbiome on low back pain (FinnGen Biobank). **(B)** Causal effect of the gut microbiome on sciatica (FinnGen Biobank) based on MR analyses.

Moreover, Figure 6 and Supplementary Table S12 demonstrated the causal effects of 196 gut microbiomes on the occurrence of back pain.

#### 3.2.6 Knee pain

The IVW test results revealed significant associations between genetically predicted relative abundance of certain bacterial taxa and the risk of knee pain. The estimates, expressed as OR with corresponding 95%CI, were as follows:

- Genus.Parabacteroides: OR = 0.972, 95% CI: 0.993 0.952, *p* = 0.01;
- Genus.Blautia: OR = 0.971, 95% CI: 0.991 0.952, *p* = 0.004.

These findings suggest a negative association between genetically influenced abundance of these bacterial taxa and the risk of knee pain. On the other hand, the genetic predisposition to knee pain was positively associated with the relative abundance of the following taxa:

- Phylum.Proteobacteria: OR = 1.02, 95% CI: 1.04 1, *p* = 0.046;
- Family.Christensenellaceae: OR = 1.023, 95% CI: 1.042 1.004, *p* = 0.017;
- Family.Rhodospirillaceae: OR = 1.013, 95% CI: 1.026 1.001, *p* = 0.039;
- Order.Rhodospirillales: OR = 1.018, 95% CI: 1.032 1.005, *p* = 0.007;
- Order.Selenomonadales: OR = 1.025, 95% CI: 1.045 1.004, *p* = 0.016;
- Class.Negativicutes: OR = 1.025, 95% CI: 1.045 1.004, *p* = 0.016;
- Genus.Anaerofilum: OR = 1.013, 95% CI: 1.024 1.002, *p* = 0.024.

**Figure 7:**
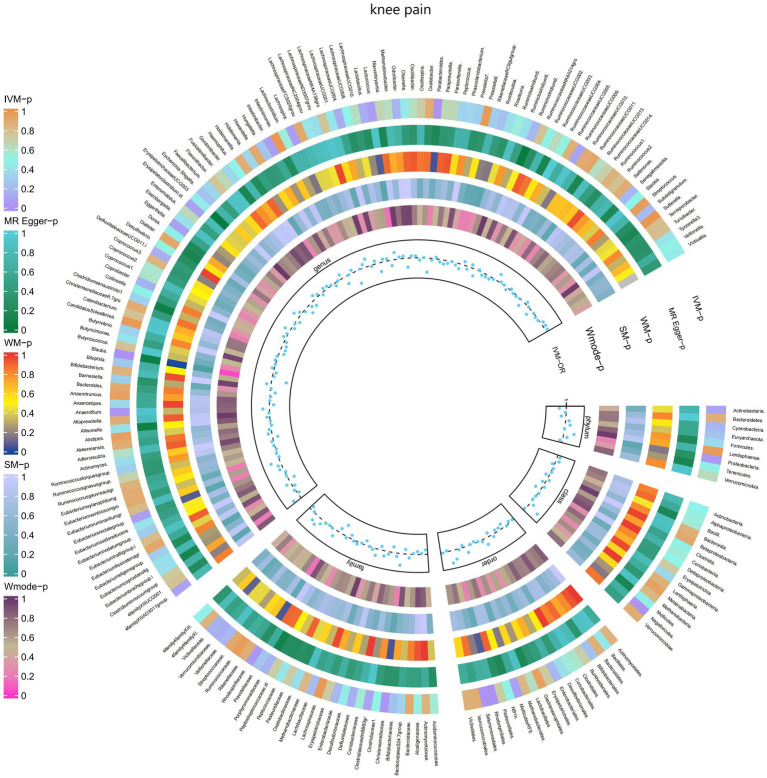
Causal effect of the gut microbiome on knee pain (UK Biobank) on MR analyses. From outside to inside, the *p*-values of IVW, MR Egger, WM, SM, and Wmode represented, respectively. IVW, inverse variance weighted; WM, weighted median; SM, simple mode; Wmode weighted mode.

Moreover, Figure 7 and Supplementary Table S13 demonstrated the causal effects of 196 gut microbiomes on the occurrence of knee pain.

#### 3.2.7 Hip pain

The IVW test results demonstrated significant associations between genetically predicted relative abundance of certain bacterial taxa and the risk of hip pain. The estimates, represented as OR with corresponding 95%CI, were as follows:

- Phylum.Tenericutes: OR = 0.976, 95% CI: 0.999 0.954, *p* = 0.042;
- Order.Bacteroidales: OR = 0.972, 95% CI: 0.996 0.948, *p* = 0.021;
- Class.Bacteroidia: OR = 0.972, 95% CI: 0.996 0.948, *p* = 0.021;
- Class.Mollicutes: OR = 0.976, 95% CI: 0.999 0.954, *p* = 0.042;
- Genus.Eisenbergiella: OR = 0.983, 95% CI: 1 0.967, *p* = 0.049.

These findings suggest a negative association between genetically influenced abundance of these bacterial taxa and the risk of hip pain. Conversely, the genetic predisposition to hip pain was positively associated with the relative abundance of the following taxa:

- Family.Porphyromonadaceae: OR = 1.036, 95% CI: 1.068 1.004, *p* = 0.027;
- Genus.Roseburia: OR = 1.033, 95% CI: 1.058 1.01, *p* = 0.005;
- Genus.Adlercreutzia: OR = 1.029, 95% CI: 1.05 1.008, *p* = 0.007.

**Figure 8:**
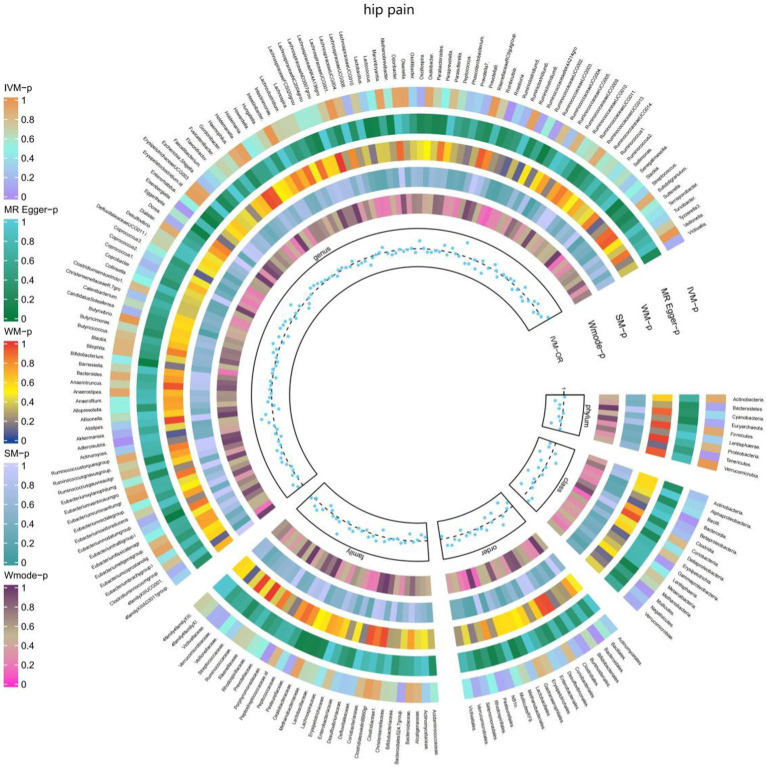
Causal effect of the gut microbiome on hip pain (UK Biobank) on MR analyses. From outside to inside, the *p-*values of IVW, MR Egger, WM, SM, and Wmode represented, respectively. IVW, inverse variance weighted; WM, weighted median; SM, simple mode; Wmode weighted mode.

Additionally, Figure 8 and Supplementary Table S14 provided evidence of the causal effects of 196 gut microbiomes on the occurrence of hip pain.

#### 3.2.8 Limb pain

The IVW test results revealed significant associations between genetically predicted relative abundance of specific bacterial taxa and the risk of limb pain. The estimates, presented as OR with corresponding 95%CI, were as follows:

- Genus.LachnospiraceaeNK4A136gro: OR = 0.919, 95% CI: 0.986 0.857, *p* = 0.019;
- Genus.RuminococcaceaeUCG003: OR = 0.919, 95% CI: 0.993 0.85, *p* = 0.032;
- Genus.Butyricicoccus: OR = 0.849, 95% CI: 0.939 0.767, *p* = 0.001;
- Genus.CandidatusSoleaferrea: OR = 0.925, 95% CI: 0.974 0.878, *p* = 0.003.

These findings indicate a negative association between genetically influenced abundance of these bacterial taxa and the risk of limb pain. On the other hand, the genetic predisposition to limb pain was positively associated with the relative abundance of the following taxa:

- Genus.LachnospiraceaeND3007grou: OR = 1.232, 95% CI: 1.49 1.018, *p* = 0.032;
- Genus.Marvinbryantia: OR = 1.116, 95% CI: 1.216 1.023, *p* = 0.013;
- Genus.Anaerotruncus: OR = 1.091, 95% CI: 1.187 1.003, *p* = 0.042.

**Figure 9:**
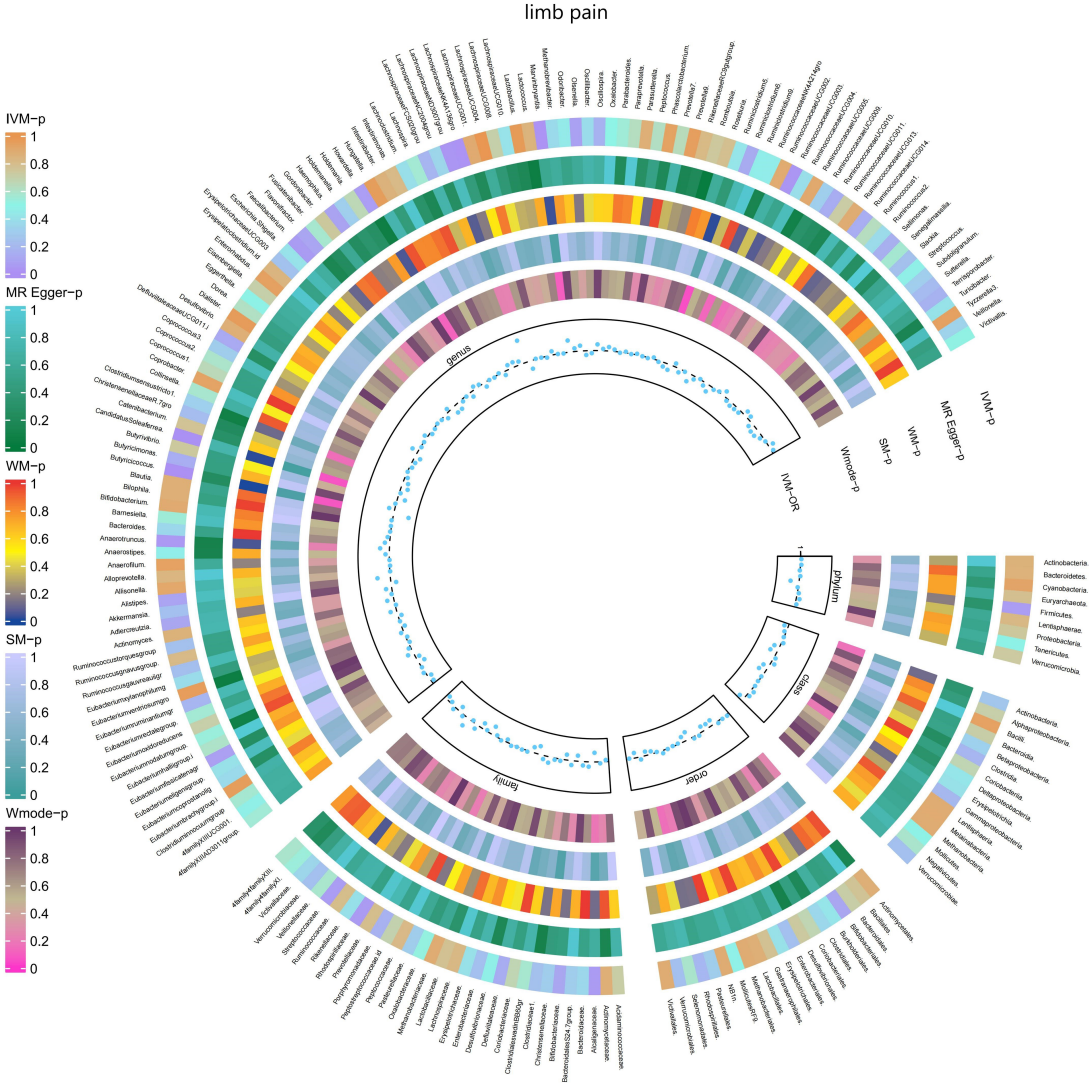
Causal effect of the gut microbiome on limb pain (FinnGen Biobank) on MR analyses. From outside to inside, the *p*-values of IVW, MR Egger, WM, SM, and Wmode represented, respectively. IVW, inverse variance weighted; WM, weighted median; SM, simple mode; Wmode weighted mode.

Furthermore, Figure 9 and Supplementary Table S15 provided evidence of the causal effects of 196 gut microbiomes on the occurrence of limb pain.

#### 3.2.9 Joint pain

The IVW test results demonstrated significant associations between genetically predicted relative abundance of certain bacterial taxa and the risk of joint pain. The estimates, presented as OR with corresponding 95%CI, were as follows:

- Genus.Terrisporobacter: OR = 0.901, 95% CI: 0.99 0.819, *p* = 0.031;
- Genus.Eubacteriumoxidoreducens: OR = 0.906, 95% CI: 0.987 0.832, *p* = 0.024.

These findings suggest a negative association between genetically influenced abundance of these bacterial taxa and the risk of joint pain. Conversely, the genetic predisposition to joint pain was positively associated with the relative abundance of the following taxa:

- Order.Selenomonadales: OR = 1.112, 95% CI: 1.229 1.006, *p* = 0.038;
- Class.Negativicutes: OR = 1.112, 95% CI: 1.229 1.006, *p* = 0.038;
- Genus.Dialister: OR = 1.11, 95% CI: 1.211 1.017, *p* = 0.019.

**Figure 10:**
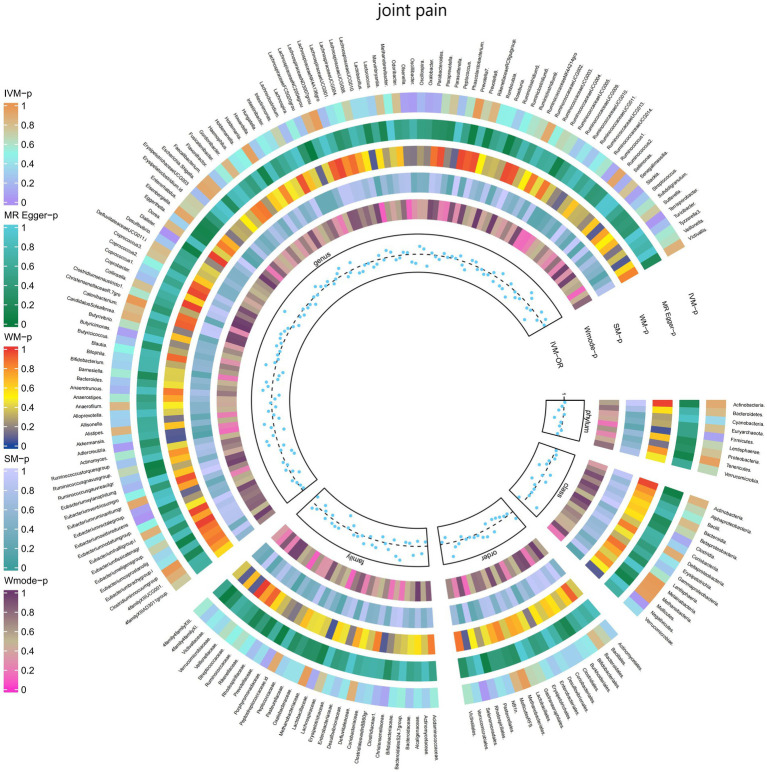
Causal effect of the gut microbiome on joint pain (FinnGen Biobank) on MR analyses. From outside to inside, the *p-*values of IVW, MR Egger, WM, SM, and Wmode represented, respectively. IVW, inverse variance weighted; WM, weighted median; SM, simple mode; Wmode weighted mode.

Furthermore, Figure 10 and Supplementary Table S16 provided evidence of the causal effects of 196 gut microbiomes on the occurrence of joint pain.

#### 3.2.10 Fibromyalgia

The IVW test results revealed significant associations between genetically predicted relative abundance of certain bacterial taxa and the risk of Fibromyalgia. The estimates, presented as OR with corresponding 95%CI, were as follows:

- Genus.Butyricicoccus: OR = 0.637, 95% CI: 0.899 0.452, *p* = 0.01;
- Genus.Erysipelatoclostridium.id: OR = 0.735, 95% CI: 0.968 0.558, *p* = 0.029.

These findings suggest a negative association between genetically influenced abundance of these bacterial taxa and the risk of Fibromyalgia. On the other hand, the genetic predisposition to Fibromyalgia was positively associated with the relative abundance of the following taxa:

- Family.Rhodospirillaceae: OR = 1.248, 95% CI: 1.552 1.003, *p* = 0.047;
- Class.Alphaproteobacteria: OR = 1.451, 95% CI: 1.943 1.084, *p* = 0.012;
- Genus.RuminococcaceaeUCG005: OR = 1.396, 95% CI: 1.781 1.095, *p* = 0.007;
- Genus.Eggerthella: OR = 1.337, 95% CI: 1.653 1.081, *p* = 0.007.

**Figure 11:**
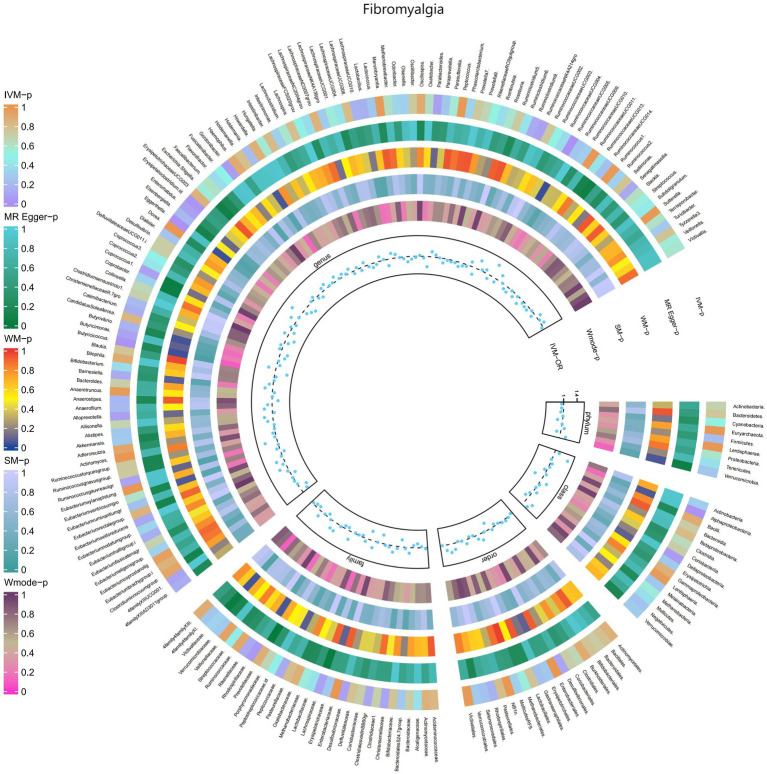
Causal efect of the gut microbiome on Fibromyalgia (FinnGen Biobank) on MR analyses. From outside to inside, the *p*-values of IVW, MR Egger, WM, SM, and Wmode represented, respectively. IVW, inverse variance weighted; WM, weighted median; SM, simple mode; Wmode weighted mode.

Furthermore, Figure 11 and Supplementary Table S17 provided evidence of the causal effects of 196 gut microbiomes on the occurrence of Fibromyalgia.

#### 3.2.11 Pain in thoracic spine

The results of the IVW test revealed significant associations between genetically predicted relative abundance of specific bacterial taxa and the risk of Pain in thoracic spine. The estimates, presented as OR with corresponding 95%CI, were as follows:

- Genus.Lachnospira: OR = 0.698, 95% CI: 0.952 0.511, *p* = 0.023;
- Genus.Eubacteriumbrachygroup: OR = 0.873, 95% CI: 0.989 0.771, *p* = 0.032.

These findings suggest a negative association between genetically influenced abundance of these bacterial taxa and the risk of Pain in thoracic spine. Conversely, the genetic predisposition to Pain in thoracic spine was positively associated with the relative abundance of the following taxa:

- Family.ClostridialesvadinBB60gr: OR = 1.279, 95% CI: 1.495 1.095, *p* = 0.002;
- Order.Bacteroidales: OR = 1.412, 95% CI: 1.761 1.132, *p* = 0.002;
- Phylum.Bacteroidetes: OR = 1.309, 95% CI: 1.712 1.001, *p* = 0.049;
- Class.Bacteroidia: OR = 1.412, 95% CI: 1.761 1.132, *p* = 0.002;
- Genus.Alistipes: OR = 1.292, 95% CI: 1.657 1.008, *p* = 0.043;
- Genus.Butyricimonas: OR = 1.193, 95% CI: 1.404 1.013, *p* = 0.034.

**Figure 12:**
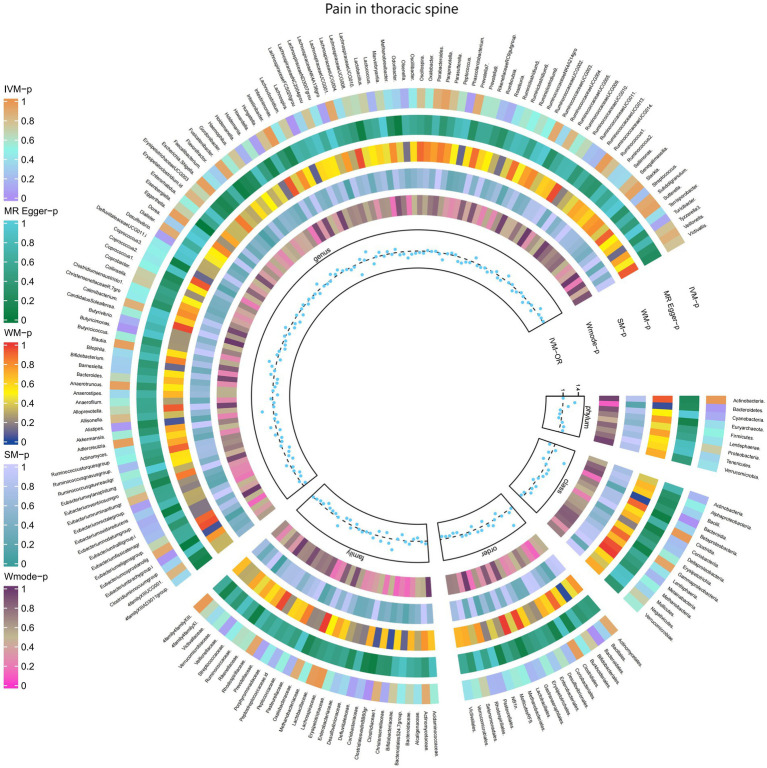
Causal effect of the gut microbiome on Pain in thoracic spine (FinnGen Biobank) on MR analyses. From outside to inside, the *p*-values of IVW, MR Egger, WM, SM, and Wmode represented, respectively. IVW, inverse variance weighted; WM, weighted median; SM, simple mode; Wmode weighted mode.

Moreover, Figure 12 and Supplementary Table S18 provided evidence of the causal effects of 196 gut microbiomes on the occurrence of Pain in thoracic spine.

#### 3.2.12 Pain (limb, back, neck, head abdominally)

The results obtained from the IVW test demonstrated significant associations between genetically predicted relative abundance of specific bacterial taxa and the risk of Pain (limb, back, neck, head abdominally). The estimates, expressed as OR with corresponding 95%CI, were as follows:

- Phylum.Firmicutes: OR = 0.936, 95% CI: 0.984 0.89, *p* = 0.01;
- Class.Clostridia: OR = 0.925, 95% CI: 0.97 0.881, *p* = 0.001;
- Class.Gammaproteobacteria: OR = 0.935, 95% CI: 0.994 0.88, *p* = 0.031;
- Class.Verrucomicrobiae: OR = 0.947, 95% CI: 1 0.897, *p* = 0.048;
- Family.Bifidobacteriaceae: OR = 0.949, 95% CI: 0.99 0.91, *p* = 0.014;
- Family.Porphyromonadaceae: OR = 0.915, 95% CI: 0.982 0.853, *p* = 0.013;
- Family.Verrucomicrobiaceae: OR = 0.947, 95% CI: 1 0.897, *p* = 0.048;
- Order.Bifidobacteriales: OR = 0.949, 95% CI: 0.99 0.91, *p* = 0.014;
- Order.Clostridiales: OR = 0.922, 95% CI: 0.968 0.877, *p* = 0.001;
- Order.Verrucomicrobiales: OR = 0.947, 95% CI: 1 0.897, *p* = 0.048;
- Genus.LachnospiraceaeNK4A136gro: OR = 0.947, 95% CI: 0.993 0.903, *p* = 0.026;
- Genus.Olsenella: OR = 0.965, 95% CI: 0.996 0.936, *p* = 0.026;
- Genus.Oscillibacter: OR = 0.962, 95% CI: 0.997 0.929, *p* = 0.032;
- Genus.RuminococcaceaeUCG011: OR = 0.955, 95% CI: 0.995 0.917, *p* = 0.027;
- Genus.Ruminococcus1: OR = 0.953, 95% CI: 0.995 0.913, *p* = 0.027;
- Genus.Akkermansia: OR = 0.947, 95% CI: 0.999 0.897, *p* = 0.047.

These findings suggest a negative association between genetically influenced abundance of these bacterial taxa and the risk of Pain (limb, back, neck, head abdominally). Conversely, the genetic predisposition to Pain (limb, back, neck, head abdominally) was positively associated with the relative abundance of the following taxa:

- Family.ClostridialesvadinBB60gr: OR = 1.279, 95% CI: 1.495 1.095, *p* = 0.002;
- Order.Selenomonadales: OR = 1.082, 95% CI: 1.149 1.018, *p* = 0.012;
- Family.Prevotellaceae: OR = 1.046, 95% CI: 1.087 1.007, *p* = 0.021;
- Class.Negativicutes: OR = 1.082, 95% CI: 1.149 1.018, *p* = 0.012;
- Genus.Eubacteriumfissicatenagr: OR = 1.036, 95% CI: 1.072 1.001, *p* = 0.041.

**Figure 13:**
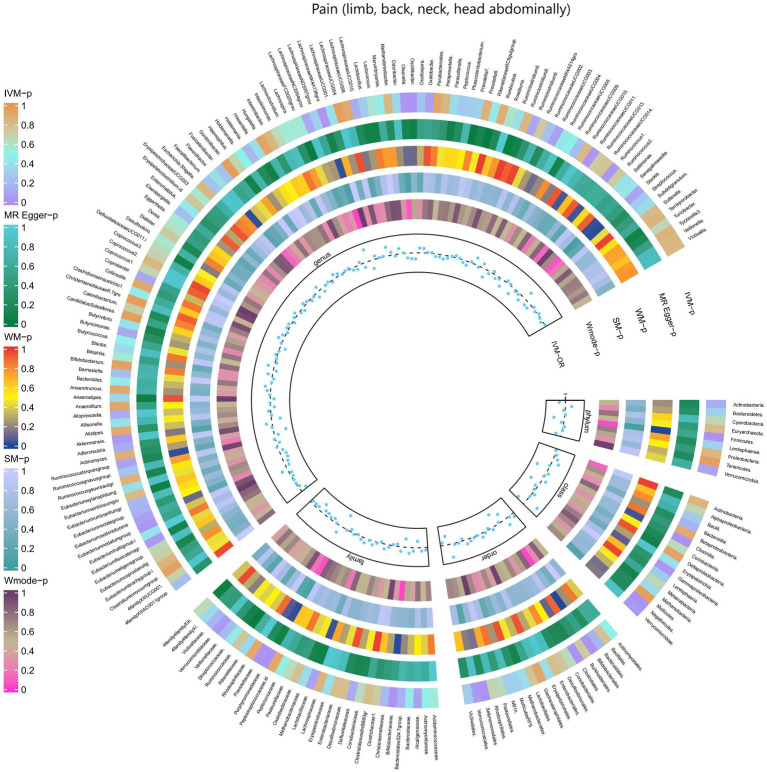
Causal effect of the gut microbiome on Pain (limb, back, neck, head abdominally) (FinnGen Biobank) on MR analyses. From outside to inside, the *p*-values of IVW, MR Egger, WM, SM, and Wmode represented, respectively. IVW, inverse variance weighted; WM, weighted median; SM, simple mode; Wmode weighted mode.

Moreover, Figure 13 and Supplementary Table S19 provided evidence of the causal effects of 196 gut microbiomes on the occurrence of Pain (limb, back, neck, head abdominally).

### 3.3 The causal associations between gut microbial metabolites and pain

The results obtained from the IVW test revealed significant associations between genetically predicted relative abundance of certain gut microbial metabolites and the risk of different types of pain. The estimates, expressed as OR with corresponding 95%CI, were as follows:5-HT: Higher genetically predicted abundance of serotonin was associated with an increased risk of limb pain (OR: 1.293, 95% CI: 1.046 1.135, *p* = 0.017). Trimethylamino oxide (TMAO): Genetically predicted higher levels of TMAO were found to be a protective factor against atypical facial pain (OR: 0.89, 95% CI: 0.804 0.975, *p* = 0.013). Glycine: Higher genetically predicted abundance of glycine was associated with an increased risk of limb pain (OR: 1.034, 95% CI: 1.004 1.066, *p* = 0.027).

However, no significant causal relationship was observed between the remaining six gut microbial metabolites and pain (as indicated in Supplementary Table S5). These findings suggest that serotonin, TMAO, and glycine play distinct roles in pain susceptibility, with serotonin and glycine being positively associated with limb pain risk, and TMAO acting as a protective factor against atypical facial pain.

### 3.4 Sensitivity analysis, Benjamini–Hochberg corrected test, Steigher test (reverse analysis)

No evidence of pleiotropic effects among the selected IVs was found in the MR-Egger analysis (*p* > 0.05) (Supplementary Table S3). Additionally, Q test from the IVW test indicated no significant heterogeneity in most causal relationships (*p* > 0.05, Supplementary Tables S4). However, when applying the Benjamini-Hochberg correction test, almost all bacterial traits did not meet the significance threshold, except for the family Bifidobacteriaceae (BH < 0.05) and the order Bifidobacteriales (BH < 0.05), which were validated in the finngen_R9_G6_MIGRAINE dataset. Furthermore, the genus Oxalobacter was found to be associated with an increased risk of low back pain, and this association remained significant even after the Benjamini-Hochberg correction was applied.

## 4 Discussion

**Figure 14:**
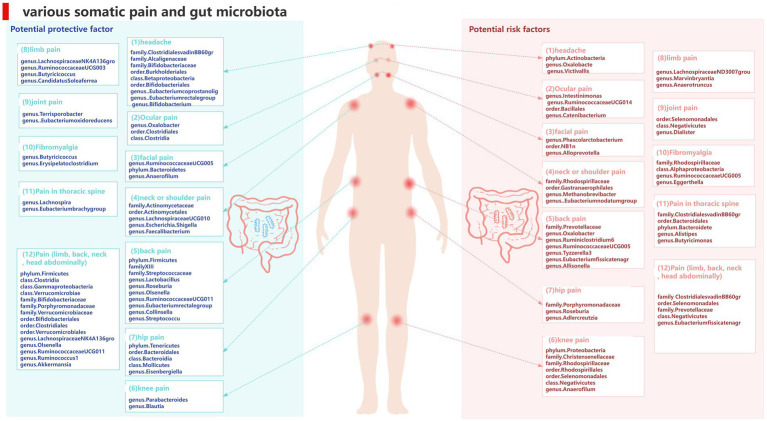
Causal links between gut microbiota and pain at 12 specific sites.

To our knowledge, this is the first study to investigate whether the gut microbiota is causally associated with chronic pain and we found evidence supporting an association between pain in specific body sites and gut microbiota, such as head, face, neck/shoulder, back, hip, knees, and limbs, and comprehensive chronic pain including Fibromyalgia, joint pain, or generalized chronic pain. We also identified metabolites of gut microbiota that could be potential risk factors for chronic pain (Figure 14). These findings may have important implications for exploring the link between gut microbiota composition and other pain conditions, identifying and isolating bacterial taxa common in general chronic pain and bacterial taxa with specific diagnoses.

More and more research findings have provided possible biological explanations for the role of gut microbiota in chronic pain (Raja et al., 2020). Gut microbiota is involved in the production of spinal cord cytokines in inflammatory pain (Amaral et al., 2008; Cohen et al., 2021). Neuronal-immune interactions (Liu et al., 2012; Shahi et al., 2017; Eccleston et al., 2021). Regulation of microglial cell activity (Erny et al., 2015, 2017; Erny and Prinz, 2020). In various mechanistic studies, bacterial metabolic end products secreted into the circulation, such as short-chain fatty acids (SCFAs) (Zhou et al., 2021) and Trimethylamino oxide (TMAO) (Koeth et al., 2013), have been shown to play a role in the generation and transmission of pain by significantly inducing oxidative stress and reducing anti-inflammatory factor levels. Interestingly, vitamin D has been found to maintain intestinal barrier integrity, suggesting a potential synergistic effect with butyrates (Murdaca et al., 2021). Additionally, SCFAs and vitamin D together enhance the synthesis of host defense peptides (HDPs), which are crucial components of the innate immune system with antimicrobial and immune-regulatory functions. This discovery opens new directions for research into the possible synergistic effects of SCFAs and vitamin D in pain mechanisms (Robinson et al., 2018).

**Figure 15:**
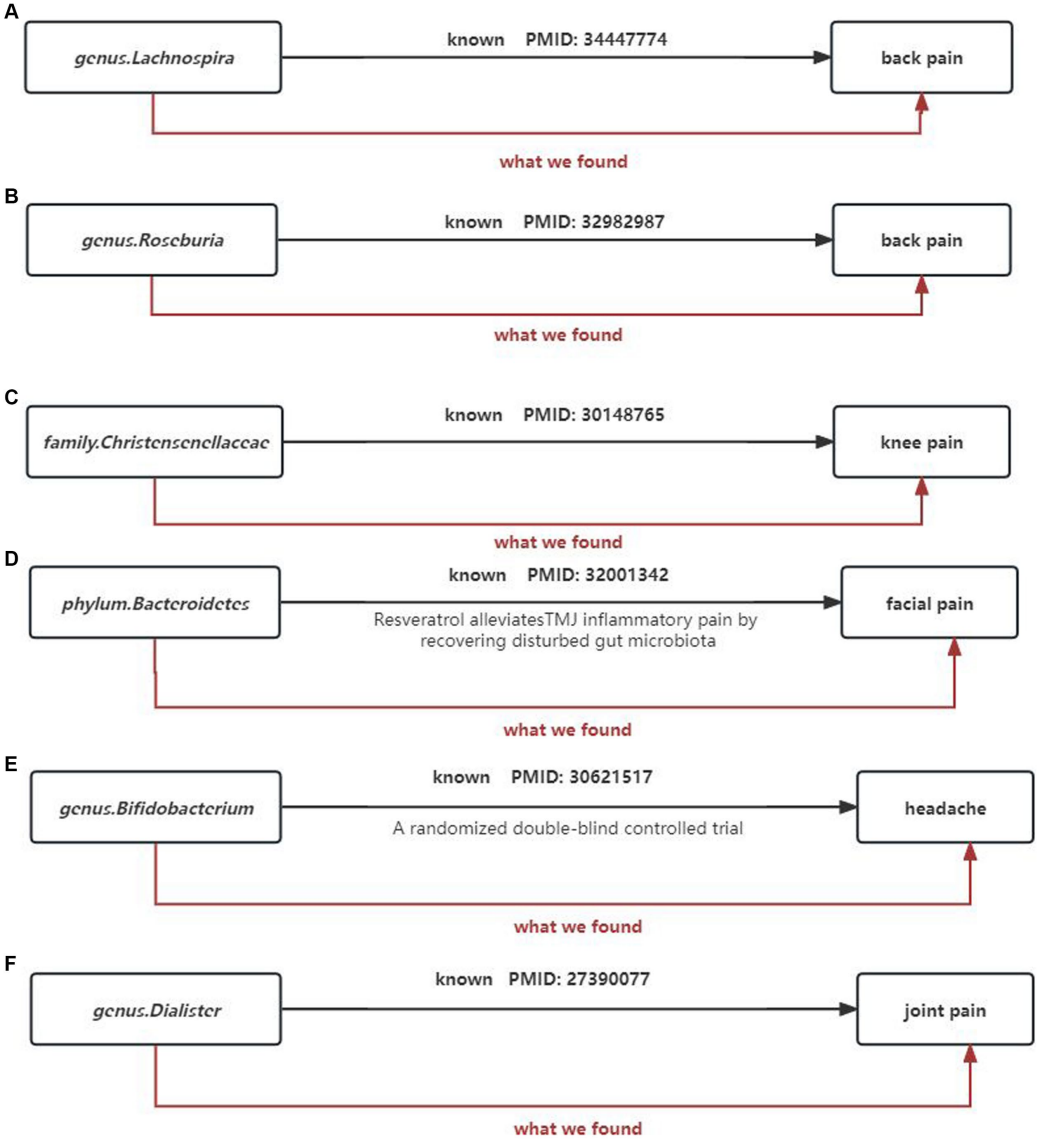
Summary of the main findings in the univariate MR Study. Combined with the available evidence, we consistently found causal effects of **(A)** genus.Lachnospira, **(B)** genus.Roseburia, **(C)** family.Christensenellaceae, **(D)** phylum.Bacteroidetes, **(E)** genus.Bifidobacterium, **(F)** genus.Dialister on painBlack solid arrows indicate known evidence and red solid arrows indicate our findings in this study.

Gut microbiota can also regulate the levels of some neurotransmitters or neuromodulators, such as GABA (Patti et al., 2012; Du et al., 2017) and serotonin (5-HT) (Yano et al., 2015), to affect neuronal function. Therefore, we not only consider the causal relationship between gut microbiota and chronic pain but also take into account the involvement of gut microbiota metabolites. We selected 9 metabolites available in the database, including 3-hydroxybutyrate (BHBA), tryptophan, tyrosine, phenylalanine, glutamate, glycine, propionic acid, TMAO, and 5-HT. In our study, 5-HT and glycine were associated with a higher risk of limb pain, while TMAO was found to be a protective factor for atypical facial pain. This is inconsistent with current research directions. In peripheral tissues, 5-HT functions as a mediator of pain and may have specific effects in various types of headaches (Cortes-Altamirano et al., 2018). However, our causal inference analysis did not provide strong evidence for a causal relationship between 5-HT and headaches, and this mechanism of pathogenesis requires further validation. At the same time, our research findings strengthen and expand existing observational evidence, indicating that gut microbiota can influence the outcomes of chronic pain (Figure 15).

Simultaneously, our research results strengthen and expand existing observational evidence, demonstrating the influence of gut microbiota on the outcomes of chronic pain (Figure 15).

(1) Our study supports genus Lactobacillus and genus Roseburia as protective factors against back pain, which is consistent with previous clinical research. Wang’s metabolomics study (Wang et al., 2021) suggests that genus Lactobacillus can alleviate abnormal inflammatory responses and improve lumbar disc herniation. Similarly, Dekker et al. (2020) also indicates that genus Roseburia is a protective factor for back pain (Figures 15A,B).
(2) The family Christensenellaceae is widely present in the human and animal gastrointestinal tract and mucosa. Studies have shown a significant correlation between the family Christensenellaceae and knee osteoarthritis as well as various musculoskeletal pains. This finding corroborates our results (Hollister et al., 2020; Figure 15C).
(3) Temporomandibular joint disorder (TMD) patients often experience persistent facial pain. A study shows that fecal microbiota transplantation with higher abundance of phylum Bacteroidetes can significantly reduce CFA-induced TMD and alleviate facial pain (Ma et al., 2020). This is consistent with the direction of our analysis results (Figure 15D).
(4) A high-quality randomized controlled trial demonstrates the beneficial effects of probiotic supplements primarily containing bifidobacteria on improving chronic headaches (Martami et al., 2019). Our analysis results corroborate that bifidobacteria are protective factors for headaches, and the results are robust (Figure 15E).
(5) Tito et al. (2017) analysis of 16S ribosomal RNA amplicon sequencing genes reveals a decrease in the abundance of genus Dialister in spondyloarthritis, while our study shows that genus Dialister is a risk factor for arthritis (Figure 15F).
(6) However, it is worth noting that for certain microbial taxa or chronic pain sites such as hip pain and neck pain, there are currently no reports on the specific effects of gut microbiota on these pain categories. Although our effect estimates show some influence, the significance of these effects disappears after FDR correction. Only the causal relationship between headaches and bifidobacteria remains strong and robust after FDR correction (FDR-corrected *p* = 0.013). A plausible hypothesis is that in addition to the gut microbiome, other factors may also influence chronic pain. Research indicates that Prebiotic Boron Complexes (PBCs), enriched with boron, foster communication between the host and its gut microbiota, aiding in the balance of beneficial and harmful bacteria within the gut (Fang et al., 2021). Moreover, PBCs bolster the intestinal barrier’s defense mechanism, preventing the infiltration of inflammatory agents into the bloodstream and thus averting persistent low-grade inflammation. This insight opens up a new perspective and lays the foundational understanding of how PBCs modulate the gut environment and their potential role in mitigating chronic pain (Fang et al., 2022).

These findings have important implications for understanding the relationship between the composition of the gut microbiome and various pain conditions. They also contribute to the identification and isolation of bacterial taxa that are commonly associated with general chronic pain, as well as specific bacterial taxa that are linked to specific pain diagnoses. It is important to note, however, that the gut microbiota is not directly involved in the transmission of bodily pain sensations, particularly those originating from the skin or limbs. The mechanisms through which the gut microbiota remotely influences bodily pain sensations remain unknown (Ma et al., 2022). Several studies have suggested fecal microbiota transplantation as a potential treatment or target for regulating neuropathic pain. Additionally, the effects of probiotic supplements and fecal microbiota transplantation on the efficacy of nonsteroidal anti-inflammatory drugs and opioid medications in pain management have been investigated (Zadori et al., 2023). These emerging insights provide new perspectives in various clinical fields, which is highly promising. Nonetheless, it is important to acknowledge that many studies are still in the preclinical stage. Further research is necessary to validate the causal relationship between chronic pain and the gut microbiota, as well as to elucidate the specific mechanisms involved.

It is important to highlight the strengths of our study. One of the key strengths is the use of MR analysis, which leverages genetic variants as proxies for environmental exposures to establish a causal relationship between exposure and disease outcome. This approach minimizes the limitations of traditional observational studies, such as residual confounding and reverse causation, by utilizing genetic differences that are assumed to be randomly assigned before birth and independent of environmental variables (Smith and Ebrahim, 2003). Furthermore, the large sample size of the GWAS employed in this study provides more precise estimates and greater statistical power.

However, it is also important to acknowledge the limitations of our study. Firstly, pain phenotyping in the UK Biobank is based on a single unified question, resulting in broadly defined and self-reported pain phenotypes that lack additional information on the nature, duration, or intensity of the pain. Similar limitations apply to blood pressure measurements. Future analyses should incorporate new, more detailed, and validated pain-related questionnaires to address these limitations. Secondly, the initial pain GWAS did not consider whether participants were taking analgesics in response to their pain questionnaire. Given that opioid receptors are present in both the digestive tract and the central nervous system, and long-term use of morphine has been associated with altered gut microbial properties, the effects of opioids on chronic pain cannot be overlooked (Banerjee et al., 2016; Acharya et al., 2017). Observational animal studies have also indicated the importance of the gut microbiota in opioid tolerance (Banerjee et al., 2016). Therefore, the effects of opioids on chronic pain cannot be disregarded. Thirdly, due to the lack of demographic data, such as gender, subgroup analysis was not feasible in the initial study. Lastly, the analysis of IVs obtained using the genome-wide statistical significance threshold of *p* < 10^−8^ was limited, only meeting full significance at the locus level (*p* < 10^−5^). These limitations restrict the generalizability of the results and may compromise the accuracy of the study.

## 5 Conclusion

In our study, we investigated the potential causal relationship between chronic pain at different body sites and the composition of the intestinal microbiota. We specifically focused on 12 body sites, including the head, face, neck/shoulder, back, hip, knee, and limbs, and various types of chronic pain such as myofibralgia, arthralgia, and generalized chronic pain. By applying rigorous statistical correction, we found that the presence of Bifidobacterium in the gut microbiota may have a protective effect against the development of chronic headaches, particularly migraine. This suggests that the gut microbial composition could serve as both biomarkers and therapeutic targets for the treatment of migraine headaches. Additionally, we identified the genus Oxalobacter as a major risk factor for back pain, and two specific metabolites produced by the intestinal microbiota as risk factors for limb pain. These findings provide valuable insights into the potential link between gut microbiome composition and different pain conditions. Furthermore, our study highlights the importance of identifying common bacterial taxa associated with general chronic pain, as well as specific bacterial taxa associated with specific pain diagnoses. Future research is needed to validate the causal relationship between chronic pain and the gut microbiota, and to elucidate the underlying mechanisms involved.

## Data availability statement

The original contributions presented in the study are included in the article/Supplementary material, further inquiries can be directed to the corresponding authors.

## Author contributions

YC: Writing – review & editing, Conceptualization, Data curation, Formal analysis, Investigation, Methodology, Resources, Software, Validation, Visualization, Writing – original draft. SW: Conceptualization, Formal analysis, Software, Validation, Writing – original draft, Writing – review & editing. JH: Validation, Writing – original draft, Writing – review & editing, Data curation, Visualization. ZW: Validation, Writing – original draft, Writing – review & editing, Resources. GH: Project administration, Writing – review & editing, Supervision. QZ: Writing – review & editing, Project administration. JZ: Project administration, Supervision, Writing – review & editing.

## Footnotes

^1^Inverse-variance weighted: The Inverse-variance weighted method synthesizes estimates of the impact of multiple genetic instrumental variables on a health outcome. This method assigns different weights to the estimates based on the variance of each instrumental variable’s effect estimate, to ensure that studies with higher precision and lower uncertainty contribute more to the final estimate. This weighting mechanism ensures that the synthesized analysis not only reflects the contributions of each independent study but also optimizes the estimate of the combined effect in a way that quantifies uncertainty.

^2^MR-Egger: The primary purpose of the MR-Egger method is to detect and correct for bias in instrumental variables, particularly directional pleiotropy (i.e., factors that affect both genetic variation and disease risk simultaneously). A key feature of this method is that it provides an intercept term, which can be used to assess the magnitude and direction of any bias present. If the intercept of the MR-Egger regression is significantly non-zero, this may indicate the presence of instrumental variable bias.

^3^Weighted Median: The Weighted Median method provides a robust effect estimate, even if some of the instrumental variables are biased. The rationale behind this method is that if more than half of the instrumental variables are valid, then the weighted median estimate will accurately reflect the true relationship between genetic variation and disease.

^4^Weighted Mode: The Weighted Mode method focuses on the most common causal estimate, i.e., those supported by the most instrumental variables. This method is particularly useful under the assumption that most instrumental variables point to the same true effect size, offering robustness against the bias of individual outlier instrumental variables.

^5^Simple Mode: The Simple Mode method is similar to the Weighted Mode but does not weight the instrumental variables. It simply identifies the most common causal effect estimate without considering the precision or uncertainty of each study result. This method may be particularly sensitive to a large number of outlier instrumental variables.

^6^*Q*-test: The *Q*-test in Mendelian randomization analysis primarily aims to assess the heterogeneity among effect estimates derived from multiple instrumental variables. The test compares the observed differences in effect sizes to what would be expected by chance alone (i.e., differences caused by sampling error), to determine whether there is statistically significant heterogeneity. If the *Q*-test indicates significant heterogeneity, this may suggest inconsistent effect estimates among different instrumental variables, prompting further exploration of potential confounding factors or biases.
